# Disseminated Histoplasmosis Presenting as Acute Respiratory Distress Syndrome and Disseminated Intravascular Coagulation in an HIV Positive Immigrant from Central America

**DOI:** 10.7759/cureus.21942

**Published:** 2022-02-05

**Authors:** Ramakanth Pata, Nway Nway, Innocent Lutaya, Victor Chen

**Affiliations:** 1 Pulmonary and Critical Care Medicine, One Brooklyn Health, New York, USA; 2 Pulmonary and Critical Care Medicine, University of Cincinnati Medical Center, Cincinatti, USA; 3 Internal Medicine, Interfaith Medical Center, New York, USA; 4 Internal Medicine, American University of Antigua, Warren, USA; 5 Department of Medicine, New York Institute of Technology College of Osteopathic Medicine, New York, USA

**Keywords:** histoplasma, immigrant, hiv, dic, ards, histoplasma capsulatum, disseminated histoplasmosis

## Abstract

Histoplasmosis rarely causes significant illness in immunocompetent patients. In endemic areas such as the Midwestern United States and Central America, most people are infected, but are rarely symptomatic, with variable presentation. The illness is usually self-limited in immunocompetent individuals. However, in immunocompromised patients, *Histoplasma capsulatum* can disseminate to various organs and should be suspected especially in the endemic areas or if there is a significant travel history involving these areas. We present a case of a 65-year-old male originally from Central America with no known past medical history presenting with Acute Respiratory Distress Syndrome complicated by disseminated intravascular coagulation due to acute histoplasmosis and incidentally found to have HIV/AIDS.

## Introduction

Histoplasmosis is one of the endemic mycoses, also known as a group of infections caused by fungi with a distant geographical distribution. Histoplasmosis is most commonly found in temperate zones worldwide; the Caribbean, Southern Mexico, Latin America, Africa, Asia, and the United States, especially in Central America and Ohio River valleys. Among the endemic mycoses, it is reportedly the most common cause of hospitalization [1]. It is asymptomatic or mildly symptomatic but self-limiting in the vast majority of cases. Some patients develop an acute pulmonary infection, referred to as Acute Histoplasmosis or in some cases, severe and disseminated infections. Disseminated histoplasmosis occurs in 1:2000 cases [2]. The severity of symptoms and the course of the disease depends on the extent of exposure and immune status of the infected individual [3]. We report a case of disseminated histoplasmosis in a patient from central America with an associated human immunodeficiency virus (HIV) infection requiring Intensive care unit (ICU) admission, whose initial presenting signs and symptoms were circulatory shock and acute respiratory distress syndrome (ARDS) complicated by disseminated intravascular coagulation (DIC).

## Case presentation

A 65-year-old male from Guatemala with an unknown past medical history presented to ED with altered mental status, generalized weakness, and decreased oral intake. At his baseline, the patient lived alone and was independent in activities of daily living (ADLs). The patient’s son noticed the patient’s acute inability to complete ADLs and intermittent confusion worsening over three days before admission leading to an unwitnessed mechanical fall without any loss of consciousness or seizure-like activity. He also reported decreased oral intake due to the feeling of “food getting stuck” in the middle of his chest, regurgitation without emesis. He denied trouble swallowing saliva and secretions but did endorse a 20-pound unintentional weight loss over a month. He also had small volume diarrhea with diffuse abdominal pain and some dyspnea three days before admission. 

**Figure 1:**
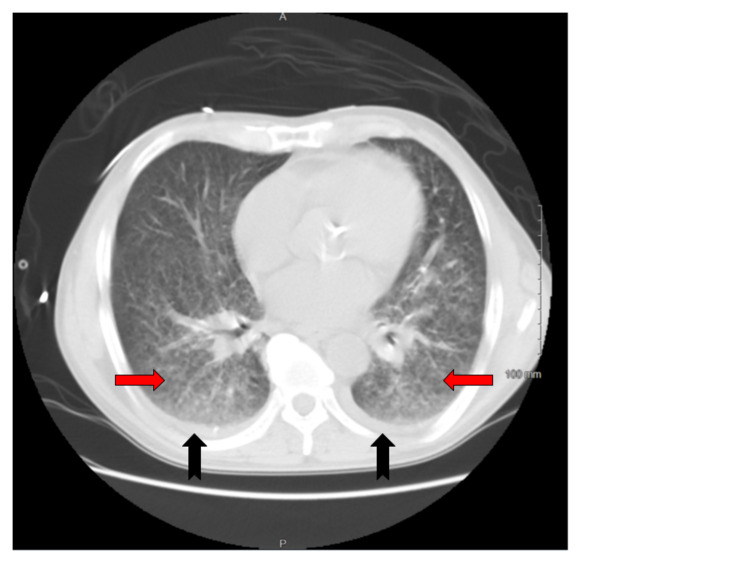
CT scan of the chest (axial view, lung window) at the level of the heart showing extensive, diffuse bilateral ground glass opacities (red arrow) without area of predominant consolidation and bilateral trace pleural effusion (black arrow), consistent with acute histoplasmosis in the patient with HIV.

**Figure 2:**
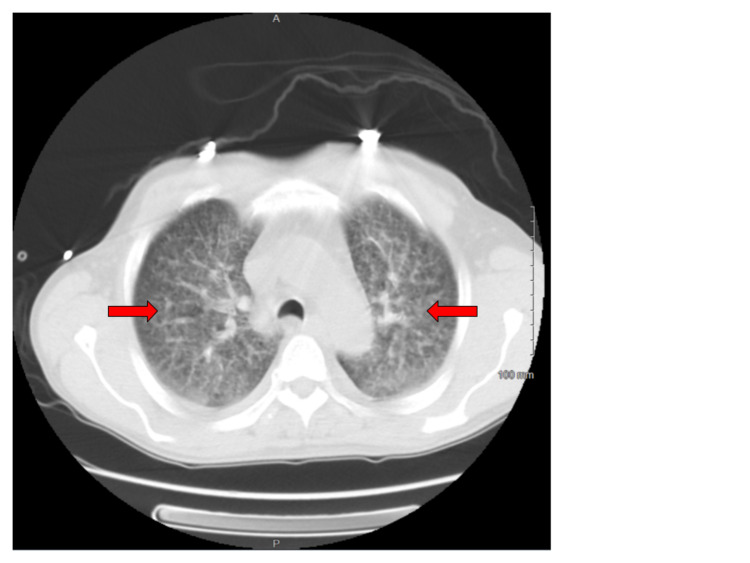
CT scan of the chest (axial view, lung window) at the level of trachea showing the extensive distribution of bilateral reticulonodular and bilateral ground glass opacities (red arrow) consistent with acute histoplasmosis in the patient with HIV.

**Figure 3:**
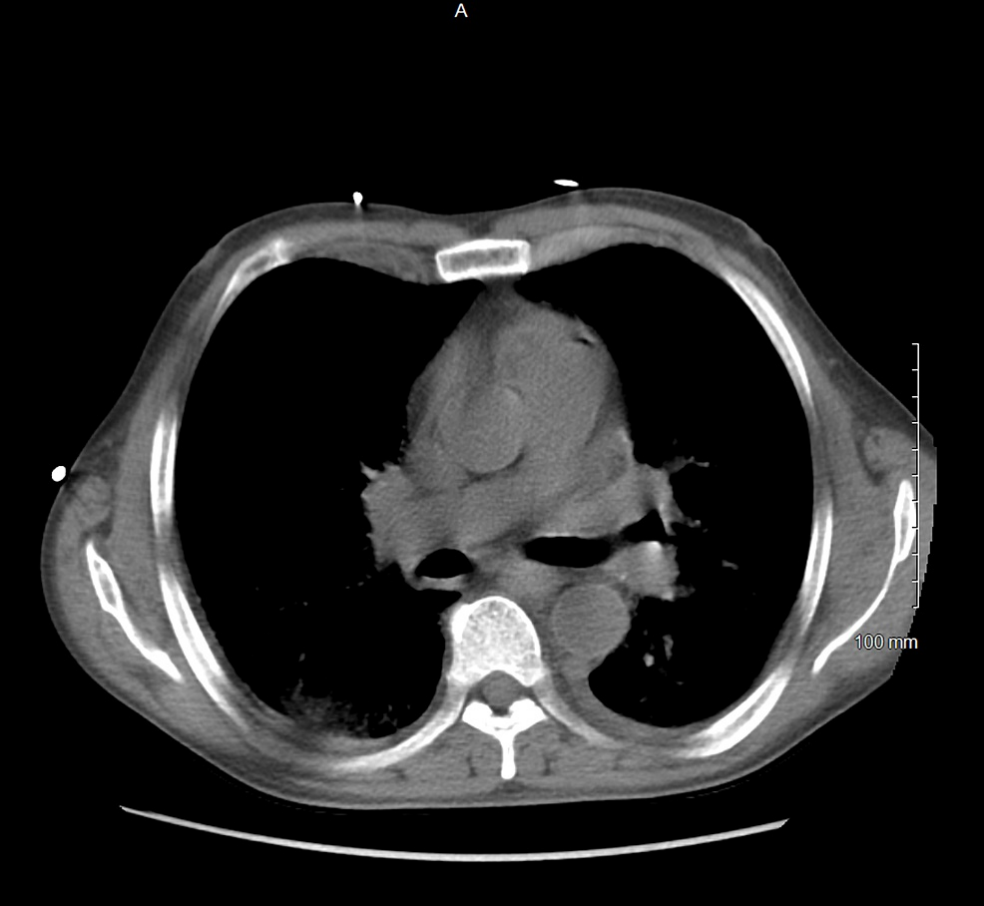
CT scan of the chest (sub carina level, mediastinal window) in the patient with acute histoplasmosis and HIV with no evidence of lymph node enlargement.

**Figure 4:**
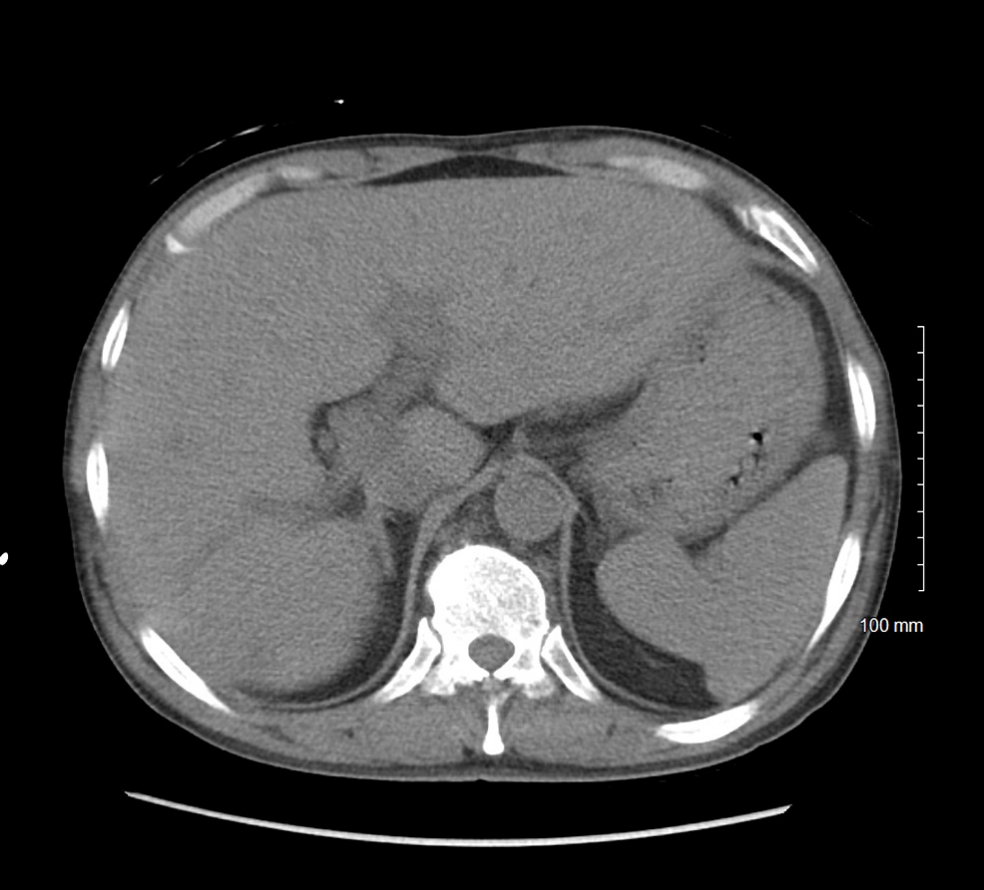
CT scan of the abdomen in the patient with acute disseminated histoplasmosis with no evidence of Liver or Spleen lesions.

On admission, he was confused, hypothermic with a core temperature of 95.5 degrees Fahrenheit, hypotensive with a blood pressure of 88/63 mm Hg, and tachypneic at a respiratory rate of 30 per minute. He was dyspneic with increased work of breathing evidenced by reciprocal abdominal breathing on 2 Liters per hour of oxygen. Some oral lesions and pharyngeal thrush were appreciated on the mouth on the ENT (ear, nose, throat) examination. He had mild crackles in bilateral lung bases. Lab results suggested acute organ dysfunction with Acute Kidney Injury, abnormal liver function tests (LFTs) associated with lactic acidosis. A presumptive diagnosis of septic shock and ARDS was considered. He was initially treated with 2.5 liters of intravenous fluids (IVF), but as the mean arterial pressure was persistently below 60 mmHg, Norepinephrine infusion was started and titrated to the target MAP (mean arterial pressure) of 65mm Hg with the addition of Vasopressin. Further workup included blood cultures, urine cultures, Computed tomography (CT) scan of the chest. CT scan of the chest showed bilateral ground-glass opacities, pulmonary edema, and trace bilateral pleural effusions with a possibility of overlying atypical pneumonia (Figures 1-3). CT scan of abdomen and pelvis revealed retroperitoneal lymphadenopathy and other findings were unremarkable (Figure 4). Broad-spectrum antibiotics with vancomycin, cefepime, metronidazole, and azithromycin were initiated. The patient was then transferred to the Medical Intensive Care Unit (MICU) for continuous monitoring and management.

The presence of oral candidiasis on physical exam prompted the search for an immunosuppressed state and he was found to be positive for HIV with a million copies and undetectable CD4 count. Empiric therapy for *Pneumocystis carinii* with Bactrim and antifungal therapy with fluconazole were started in addition to broad-spectrum antibiotics.

Within 72 hours of admission, his hypoxia worsened requiring intubation, secondary to Acute Respiratory distress syndrome. Multiorgan dysfunction on day two of admission included acute kidney injury and acute liver failure requiring continuous renal replacement therapy (CRRT) with an overall SOFA (sequential organ failure assessment) score of 12. Given the poor response to empiric therapy for 72 hours and the atypical appearance of CT chest for *Pneumocystis Jirovecii* infection, further workups were warranted for fungal blood cultures, fungal antigen testing, cytomegalovirus, and Epstein Barr virus. He was also noted to have disseminated intravascular coagulation with thrombocytopenia, dysfibrinogenemia, abnormal thromboelastogram requiring multiple rounds of packed red cells cryoprecipitates, and fresh frozen plasma.

Bronchoalveolar lavage from Bronchoscopy was sent for culture, which was negative for respiratory viral panels including influenza, COVID, Pneumocystis pneumonia and cryptococcus, bacterial culture, and AFB (acid-fast bacilli). Urine legionella and Pneumococcal antigen were negative. However, blood and BAL (bronchoalveolar lavage) smear were concerning for Histoplasmosis and empiric Liposomal amphotericin was started. Further workup revealed a serum histoplasmosis antigen level of greater than 20 (ref range 1:8), Fungitell (1,3 ß-D glucan) of 198 (ref range: 10 to 40 pg/mL). Given his pulmonary disease, hepatic dysfunction, concerning blood smear, and positive blood culture after ruling out most other infectious etiologies, the patient was diagnosed with disseminated histoplasmosis in liaison with the infectious disease team. There was a dramatic improvement in hemodynamic status with recovering organ function and was extubated on day seven of admission with normalization of liver and kidney function. Amphotericin was given for two weeks and switched to Itraconazole. The plan was to continue therapy for one year, with serum level monitoring every two weeks. Highly active antiretroviral therapy (HAART) for HIV was started approximately around the 10th day of admission, including prophylaxis for Pneumocystis and Toxoplasma. The patient was discharged to short-term rehab for ICU-acquired weakness.

## Discussion

Literature on Histoplasmosis first occurred in 1906 by a US army doctor [4]. To date, it is reported to be the most prevalent endemic mycotic infection in the United States [1]. In the United States, it's usually located within the Midwestern and Central states, and most commonly along the Ohio and Mississippi River Valleys. About half a million cases are reported in the United States every year [5]. Increasing reports of histoplasmosis in HIV-positive patients from Latin America are being documented [6-7]. It is therefore important to integrate and screen immigrants with risk factors for HIV status. *Histoplasma capsulatum* grows best in soil contaminated with bird or bat droppings. This is thought to be due to the alteration of the soil by the droppings that increase nitrogen, which in turn favors sporulation. Therefore, the major form of spread of *H. capsulatum* is via spore inhalation. Common sites include farms, abandoned buildings, and caves [8]. Activities that put individuals at great risk of exposure include construction, building demolition, cave exploring, cleaning bird or bat droppings, or any activities that disrupt the droppings that were initially enclosed in an area [9-11].

Histoplasmosis infections tend to be asymptomatic and self-limited; however, a few individuals develop acute pulmonary pneumonia. The worst cases are usually characterized by a progressive and severe disseminated form of the infection [2]. Acute diffuse pulmonary histoplasmosis presents with diffuse reticulonodular pulmonary infiltrates, which can progress to respiratory failure like the patient in this case [12]. Patients with underlying lung disease are at risk of the development of chronic pulmonary histoplasmosis characterized by persistent cavitation, pulmonary fibrosis, and progressive pulmonary insufficiency [13]. After years of initial infection, calcification of peribronchial lymph nodes and pulmonary granulomas develops. This leads to broncholithiasis, which may erode into adjacent bronchi causing expectoration of small stones [14,15]. Primary histoplasmosis can be healed by mediastinal fibrosis, which is the most serious late complication. It is characterized by a dense fibrous capsule that surrounds a mediastinal area of old caseous lymph nodes extending within the lumen of critical mediastinal structures leading to complete occlusion [16, 17].

Histoplasmosis spreads rapidly and hematogenously in an acute setting. This usually occurs before the immunity is mounted against it. This puts immunocompromised patients such as HIV-positive persons, older patients, transplant patients, and patients on TNF-alpha inhibitors at particular risk of extrapulmonary dissemination. Disseminated histoplasmosis occurs in about 1 of 2000 patients with acute histoplasmosis [3, 18, 19]. The disseminated form of *H. capsulatum* presents with symptoms affecting almost all body systems including but not limited to skin, gastrointestinal, adrenal, central nervous system, and heart involvement. [11]. Signs and symptoms can include encephalitis, meningitis, focal brain symptoms, endocarditis, adrenal insufficiency, and many more per the involved system [20]. It is important to consider disseminated histoplasmosis in immigrant patients with unknown HIV positive or medical status if the above symptoms are observed. 

The acute infection manifests as fevers, chills, fatigue, pancytopenia due to bone marrow involvement, and hepatosplenomegaly. Gastrointestinal symptoms such as diarrhea are rare. Patients with AIDS and those who are severely immunocompromised due to immunosuppressive medication usually manifest with shock, renal failure, hepatic failure, respiratory distress, and coagulopathies [3]. For this reason, disseminated histoplasmosis should be considered in all newly diagnosed patients with undifferentiated shock, and HAART therapy should be started immediately. The patient in our case presented with hemodynamic instability with circulatory shock, renal failure, hepatic failure, ARDS, and hypothermia. The patient was found to have an HIV infection that takes the lead in the diagnosis of disseminated histoplasmosis. Therefore, early initiation of the HARRT and amphotericin B steered into clinical recovery.

## Conclusions

Pulmonary infection is the most common presentation of Histoplasmosis as it is transmitted via inhalation of the spores. Histoplasmosis should also be considered in patients with newly diagnosed HIV with undifferentiated shock, ARDS, and DIC. It is imperative to recognize the origin of the immigrant when considering the diagnosis. Geographic distribution plays a major role in determining how and where patients acquire endemic mycoses such as *H. capsulatum*. Immigrant patients who are usually unknown to the medical database systems tend to miss out on prompt diagnosis, hence delaying treatment. This can lead to an unfavorable prognosis of Histoplasmosis.

## References

[1] Chu JH, Feudtner C, Heydon K, Walsh TJ, Zaoutis TE (2006). Hospitalizations for endemic mycoses: a population-based national study. Clin Infect Dis.

[2] Kauffman CA (2007). Histoplasmosis: a clinical and laboratory update. Clin Microbiol Rev.

[3] Wheat LJ, Connolly-Stringfield PA, Baker RL (1990). Disseminated histoplasmosis in the acquired immune deficiency syndrome: clinical findings, diagnosis and treatment, and review of the literature. Medicine (Baltimore.

[4] Darling ST (1906). A protozoan general infection producing pseudotubercles in the lungs and focal necrosis in the liver, spleen and lymph nodes. JAMA.

[5] Hammerman KJ, Powell KE, Tosh FE (1974). The incidence of hospitalized cases of systemic mycotic infections. Sabouraudia.

[6] Adenis AA, Valdes A, Cropet C (2018). Burden of HIV-associated histoplasmosis compared with tuberculosis in Latin America: a modelling study. Lancet Infect Dis.

[7] Samayoa B, Roy M, Cleveland AA (2017). High Mortality and Coinfection in a Prospective Cohort of Human Immunodeficiency Virus/Acquired Immune Deficiency Syndrome Patients with Histoplasmosis in Guatemala. Am J Trop Med Hyg.

[8] Benedict K, Mody RK (2016). Epidemiology of Histoplasmosis Outbreaks, United States, 1938-2013. Emerg Infect Dis.

[9] Centers for Disease Control and Prevention (CDC) (2014). Histoplasmosis outbreak associated with the renovation of an old house - Quebec, Canada, 2013. MMWR Morb Mortal Wkly Rep.

[10] Ward JI, Weeks M, Allen D (1979). Acute histoplasmosis: clinical, epidemiologic and serologic findings of an outbreak associated with exposure to a fallen tree. Am J Med.

[11] Wheat LJ, Conces D, Allen SD, Blue-Hnidy D, Loyd J (2004). Pulmonary histoplasmosis syndromes: recognition, diagnosis, and management. Semin Respir Crit Care Med.

[12] Kataria YP, Campbell PB, Burlingham BT (1981). Acute pulmonary histoplasmosis presenting as adult respiratory distress syndrome: effect of therapy on clinical and laboratory features. South Med J.

[13] Wheat LJ, Wass J, Norton J, Kohler RB, French ML (1984). Cavitary histoplasmosis occurring during two large urban outbreaks. Analysis of clinical, epidemiologic, roentgenographic, and laboratory features. Medicine (Baltimore).

[14] Conces DJ Jr, Tarver RD, Vix VA (1991). Broncholithiasis: CT features in 15 patients. AJR Am J Roentgenol.

[15] Shah SS, Karnak D, Shah SN, Budev M, Machuzak M, Gildea TR, Mehta AC (2007). Broncholith caused by donor-acquired histoplasmosis in a lung transplant recipient. J Heart Lung Transplant.

[16] Goodwin RA, Nickell JA, Des Prez RM (1972). Mediastinal fibrosis complicating healed primary histoplasmosis and tuberculosis. Medicine (Baltimore).

[17] Loyd JE, Tillman BF, Atkinson JB, Des Prez RM (1988). Mediastinal fibrosis complicating histoplasmosis. Medicine (Baltimore).

[18] Davies SF, Khan M, Sarosi GA (1978). Disseminated histoplasmosis in immunologically suppressed patients. Occurrence in a nonendemic area. Am J Med.

[19] Smith DK, Neal JJ, Holmberg SD (1993). Unexplained opportunistic infections and CD4+ T-lymphocytopenia without HIV infection. An investigation of cases in the United States. The Centers for Disease Control Idiopathic CD4+ T-lymphocytopenia Task Force. N Engl J Med.

[20] Wheat LJ, Musial CE, Jenny-Avital E (2005). Diagnosis and management of central nervous system histoplasmosis. Clin Infect Dis.

